# The Impact of Social Determinants of Health on Ocular Diseases in Western New York: A Comparative Ecological Study of Two U.S. Counties

**DOI:** 10.3390/healthcare13233089

**Published:** 2025-11-27

**Authors:** Abdullah Virk, Henry Qin, Mohammed Mehdi Shahid, Honghong Liu, Changyong Feng, Karen Allison

**Affiliations:** 1 College of Medicine-Phoenix, University of Arizona, Phoenix, AZ 85004, USA; azvirk@arizona.edu; 2 Flaum Eye Institute, University of Rochester, Rochester, NY 14641, USA; henry_qin@urmc.rochester.edu (H.Q.); mehdi_shahid@urmc.rochester.edu (M.M.S.); 3 Department of Biostatistics and Computational Biology, University of Rochester, Rochester, NY 14642, USA; honghong_lin@urmc.rochester.edu (H.L.); changyong_feng@urmc.rochester.edu (C.F.)

**Keywords:** social determinants, ocular disease, public health

## Abstract

**Background/Objectives:** As the world becomes more connected, it is becoming critical for clinicians to understand other cultures, races, and ethnicities to provide the most effective therapy. A comprehensive understanding of all communities necessitates an examination of the social determinants of health (SDH). Eye diseases and many other conditions are influenced by SDH. To elucidate the impact of SDH on eye health, a comparative ecological analysis of Monroe and Erie Counties in New York State was conducted to identify any differences in SDH and glaucoma, age-related macular degeneration (AMD), and diabetic retinopathy prevalence. **Methods**: The CDC Vision and Eye Health Surveillance System (VEHSS) was utilized to collect glaucoma, AMD, and diabetic retinopathy data in Monroe and Erie Counties, New York State, and the USA for 2019. The American Community Survey, County Health Rankings, and Neighborhood Atlas data portals were used to collect the county socioeconomic demographics, along with other health statistics. **Results:** Overall, Erie County had a higher prevalence of AMD (9.56% vs. 6.61%, *p* < 0.0001) and glaucoma (13.05% vs. 11.71%, *p* < 0.0001) compared to Monroe County. Erie County also had a higher prevalence of AMD and glaucoma across all races, aside from North American Natives. Erie County also had a greater primary care shortage, with only 1 primary care physician for every 1230 individuals. Although income inequality and poverty were similar between Erie and Monroe Counties, Erie County also has more racial segregation regarding the residential layout (ranked 74 on a scale of 0 to 100, with 100 being the most segregated). **Conclusions:** The results indicate that Erie County had an increased prevalence of AMD and glaucoma compared to Monroe County in 2019, along with a greater primary care shortage. Although this analysis targeted Western New York, disparities such as lack of primary care access and segregation are prevalent across the US, necessitating widespread action to address these problems.

## 1. Introduction

With the growing global interconnectedness and the increasing diversity of patient populations, especially in countries like the US, it is becoming essential for clinicians to understand cultures, races, and ethnicities in order to provide adequate and personalized healthcare delivery for their patients. One area that must be addressed is the impact of social determinants of health (SDH) on healthcare. The World Health Organization (WHO) defines social determinants of health as non-medical factors that influence health outcomes, such as conditions in which people are born, grow up, work, live, and age [1]. These social determinants impact a person’s quality of life by influencing their susceptibility to develop a disease, ability to receive adequate treatment, and tendency to be compliant with care and build patient–provider trust. The health impact of these determinants ranges from long-term to short-term health risks. Understanding what social determinants are and how to address them during patient care is a vital skill needed in the clinical field, not only to provide more personalized care but also to build better relationships with patients and achieve better long-term outcomes. To understand the impact of certain social determinants on eye health, a comparative analysis was conducted to identify any differences in social determinants of health and in glaucoma, age-related macular degeneration (AMD), and diabetic retinopathy prevalence in Monroe and Erie Counties, New York State.

### 1.1. Social Determinants

**Figure 1:**
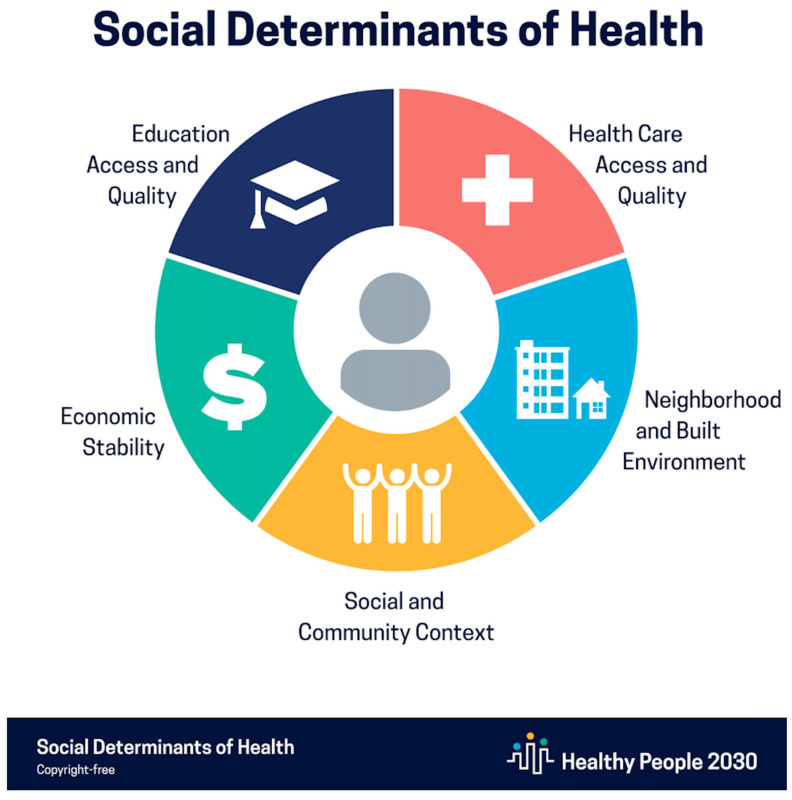
Social determinants of health. Healthy People 2030, US Department of Health and Human Services, Office of Disease Prevention and Health Promotion [2].

According to the US Department of Health and Human Services, social determinants of health (SDH) can be grouped into 5 main categories: economic stability, education access and quality, healthcare access and quality, neighborhood and built environment, and social and community context (Figure 1) [2]. Economic stability includes factors such as employment, food security, housing instability, and decreasing poverty. The education access and quality category includes childhood development and education, higher education, language, and literacy. The healthcare access and quality category comprises health services, such as access to primary and specialist care, and cost of care, including health insurance availability. Additionally, the neighborhood and built environment category incorporates the availability of healthy foods, the presence of crime and violence, environmental conditions, and quality of housing. Lastly, the social and community category incorporates factors such as civic participation, discrimination, and incarceration [2]. Each category encompasses various other factors that can play a role in an individual’s health outcomes. Moreover, the World Health Organization (WHO) emphasized a common theme present in many countries: the lower a person’s socioeconomic status, the worse their health [1]. These determinants play a crucial role in the modern landscape of health disparities, while also highlighting the continued side effects of the historical maltreatment of minority communities.

### 1.2. Glaucoma

Glaucoma is a neurodegenerative disorder that affects the optic nerve, resulting in progressive vision loss. Typically, individuals of older age are more susceptible to glaucoma. A family history of glaucoma may increase an individual’s risk of developing the disease [3]. Intraocular pressure (IOP) is the only modifiable risk factor for glaucoma. High IOP (usually above 21 mmHg) can result in progressive damage to the optic nerve. One major risk factor found to affect the risk of glaucoma is race, with minorities such as Black/African American and Hispanic/Latino individuals being more vulnerable to this disease [3,4]. In the Ocular Hypertension Treatment Study, a prospective study with 1636 patients, the 20-year cumulative incidence of glaucoma was much higher among individuals of Black race (55.2%) when compared independently to all other races (47.2%) [5]. Acuff et al. analyzed almost 3000 glaucoma patients using the NIH All of Us database and found that African American participants and participants of other racial minorities were diagnosed with glaucoma at a mean age of 60 years old, which is earlier than that of White participants with a mean age of 66 years old. A similar result was found when analyzing the mean glaucoma diagnosis age of Hispanic or Latino patients compared to non-Hispanic or Latino White participants (mean 61 vs. 65 years) [5]. Overall, the higher prevalence and risk of having a diagnosis of glaucoma leading to blindness in Blacks or Hispanics compared with other races, particularly Caucasians, may be indicative of the systematic disadvantages of these populations. Populations who are marginalized and historically ignored have fewer resources and less health literacy to seek preventive medical treatment, such as eye screenings and exams, before actual symptoms occur. When some patients finally do seek medical attention, glaucoma symptoms are quickly escalating, with rapid loss of vision already occurring. Thus, it remains critical to foster publicly funded programs that increase access to prevention and screening programs for disadvantaged populations.

### 1.3. Diabetes

Diabetes mellitus is a metabolic disease that affects the body’s ability to control blood glucose, leading to elevated blood sugar levels. The two main categories of diabetes are type 1 and type 2. Type 1 diabetes mellitus (T1DM) results in the pancreas being unable to produce insulin, which is a hormone that helps regulate blood glucose levels. Type 2 diabetes mellitus (T2DM) results in the loss of the ability of insulin to act on insulin receptors [6]. Both types of diabetes cause an increase in blood glucose levels, either due to lack of insulin or insulin resistance. According to the CDC, the prevalence of diabetes in the United States in 2021 was 11.6%, which is approximately 38.4 million people. Data from 2019 to 2021 showed that the prevalence of diagnosed diabetes among adults was highest for American Indians and Alaska Natives at 13.6%, then non-Hispanic Blacks at 12.1%, Hispanics at 11.7%, Asians at 9.1%, and non-Hispanic Whites at 6.9% [7].

Both T1DM and T2DM are risk factors for developing diabetic retinopathy [8]. Long-term diabetes that is not controlled can cause ocular pathology and disorders such as diabetic retinopathy (DR). DR results from the high blood glucose levels, which may cause damage to the retina by blocking blood flow in the arteries and capillaries that deliver nutrients to the retina [9]. These blockages may lead to protrusions of the blood vessels or dilation of the blood vessels. Over time, if blood glucose levels are not managed, it can lead to proliferative DR, where new abnormal blood vessels start growing, which further damages the retina. These blood vessels are often weak and prone to leaking, which results in edema in the retina as well as blood in the vitreous, leading to symptoms such as decreased vision and vision loss [9].

### 1.4. Age-Related Macular Degeneration

Age-related Macular Degeneration (AMD) is another leading cause of irreversible blindness worldwide. In the United States, approximately 20 million people have AMD. There are several risk factors associated with this disease [10]. Non-modifiable risk factors include gender, ethnicity, and genetic predisposition. One of the predominant modifiable risk factors is smoking, which has been shown to increase the risk for AMD by 2–4 fold [11]. Quitting smoking for the long term has been shown to decrease the risk of AMD. Other modifiable risk factors include diet, body mass index, physical activity, and sunlight exposure. Oxidative stress is another component that has been associated with an increased risk for AMD due to molecules such as reactive oxygen species (ROS) and reactive nitrogen species (RNS) that damage cells. Antioxidants, therefore, can be used to reduce the formation of ROS and RNS. Antioxidants include vitamins (vitamin C and vitamin E), carotenoids, and elements such as zinc. Additionally, regular physical activity has been shown to increase antioxidant enzyme activity [11].

Common ocular diseases such as glaucoma, age-related macular degeneration, and diabetic retinopathy are often a result of a multitude of factors. This study aims to elucidate the population-level impact of various social determinants on ocular health in Western New York using an ecological design. Diseases such as glaucoma, diabetic retinopathy, and age-related macular degeneration will be compared between Erie and Monroe Counties.

## 2. Methods

This descriptive ecological study aimed to understand the effect of the social determinants of health on ocular diseases and formulate methods to assist with addressing these disparities. To analyze the effect of social determinants on eye diseases, the CDC’s publicly available Vision and Eye Health Surveillance System (VEHSS) was used to collect glaucoma, AMD, and diabetic retinopathy prevalence data with 95% confidence intervals in Monroe and Erie Counties, New York State, and the USA for 2019. The year 2019 had the most stratified data based on age, race, and gender compared to other years. VEHSS is a CDC website that uses existing data sources to help healthcare professionals and policymakers understand eye disorders, vision loss, and eye care services around the country, states, and counties. By using Medicare as the primary data source, VEHSS contains composite prevalence estimates for vision impairment, blindness, and other ocular diseases with the aim of highlighting health disparities in visual treatment and outcomes, prevalence of ocular disorders, and the use of eye health services [12].

The American Community Survey and County Health Rankings data portals were used to collect the socioeconomic demographics of the counties of interest, specifically poverty rates, income inequality, healthcare access, and educational attainment, along with other county health statistics for the year 2019. County Health Rankings and Roadmaps, which is a program of the University of Wisconsin Population Health Institute, highlights various policies and practices to improve the health of people. In addition, the website provides a snapshot of health variables of nearly every county in the United States, such as insurance rates, opportunities for education, and safe housing to allow for community efforts to address core community health problems [13]. The American Community Survey, which is conducted by the US Census Bureau, is an annual survey that collects information about the demographic, economic, social, and housing characteristics of the nation, states, and individual counties. This survey aims to help local officials and community leaders understand changes in their community to make more informed decisions when addressing issues [14].

To create a graphical representation of the socioeconomic disparities within each county, Area Deprivation Index (ADI) data for 2022 was also collected from the Neighborhood Atlas Portal by the Center of Health Disparities Research at the University of Wisconsin School of Medicine and Public Health. The Neighborhood Atlas aims to publicly share measures of neighborhood disadvantage to inform future health delivery and policy. The ADI provided by the Neighborhood Atlas allows for rankings of neighborhoods by socioeconomic disadvantage around the US at a national and state level. Data can be sorted and downloaded via 12-digit FIPS codes or 9-digit ZIP codes. These rankings are determined by a plethora of factors such as income, education, employment, and housing quality [15,16]. Data for ADI was most recently provided in 2022 and was not available for the year 2019. The closest available data was 2020, which was significantly impacted by the COVID-19 pandemic, resulting in the most recent available year, 2022, being chosen.

For each continuous outcome variable, two-sample *t*-tests were used to compare the mean values of two counties. For each categorical variable, Pearson’s chi-square test was used to compare the distributions between two counties. The significance level was set at 0.05 for each analysis. All analyses were implemented with SAS 9.4 (SAS Institute Inc., Cary, NC, USA).

## 3. Results

**Figure 2:**
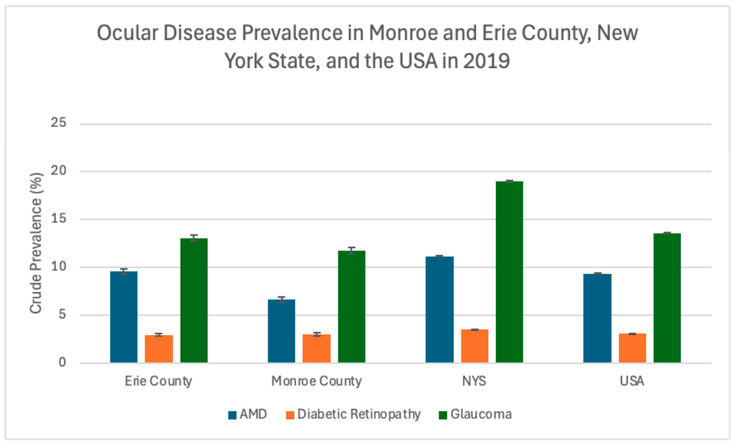
Ocular disease crude prevalence in Monroe and Erie Counties, New York State (NYS), and the USA in 2019. Error bars represent 95% confidence intervals.

According to the US Census Bureau American Community Survey, the total population for the USA and the state of New York in 2019 was estimated to be 328,239,523 and 19,453,561, respectively. The population estimates for Erie and Monroe Counties, New York, were 918,702 and 741,770, respectively [17]. Glaucoma, Age-Related Macular Degeneration (AMD), and Diabetic Retinopathy (DR) prevalence data for the year 2019 are summarized in Table 1, Table 2, Table 3, Table 4 and Table 5. Erie County had a higher prevalence of AMD (9.56% vs. 6.61%, *p* < 0.0001) and glaucoma (13.05% vs. 11.71%, *p* < 0.0001) compared to Monroe County (Table 1). However, New York State had higher AMD (11.18%), DR (3.55%), and glaucoma (19.00%) prevalence compared to both counties and US estimates (Table 2 and Figure 2). Sample sizes of Monroe County, Erie County, and New York State (NYS) are noted for Table 1 and Table 2, signifying the total population per location available in the Medicare data used by VEHSS.

**Figure 3:**
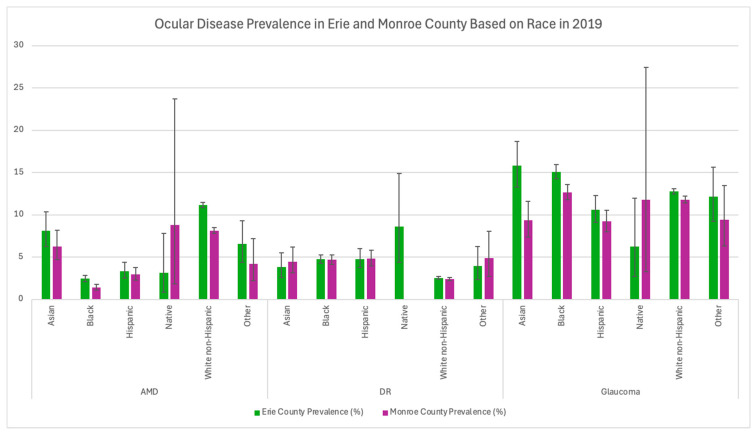
Ocular disease prevalence in Monroe and Erie Counties based on race in 2019. Error bars represent 95% confidence intervals.

**Figure 4:**
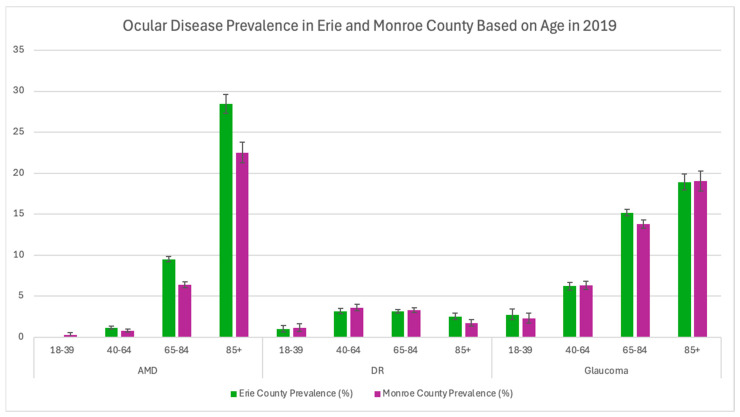
Ocular disease prevalence in Monroe and Erie Counties based on age in 2019. Error bars represent 95% confidence intervals.

Additionally, Erie County had greater AMD and glaucoma prevalence across all races except for North American Natives (Table 3 and Figure 3). In Erie County, Black and Asian populations had the highest prevalence of glaucoma at 15.08% and 15.84%, respectively. Moreover, diabetic retinopathy prevalence between Black and Hispanic populations in Erie and Monroe Counties was very similar (Table 3 and Figure 3). In 2019, Erie County had higher prevalence rates for males (7.42% vs. 4.99%) and females (11.39% vs. 7.96%) with AMD compared to Monroe County (*p* < 0.0001). Additionally, Erie County had a higher prevalence of males (11.79% vs. 10.75%, *p* = 0.002) and females (14.13% vs. 12.52%, *p* < 0.0001) with glaucoma as well (Table 4). Females in both counties had a higher prevalence of AMD and glaucoma compared to males. Across all available age data, Erie County also had a higher prevalence of AMD (*p* < 0.005) compared to Monroe County (Table 5 and Figure 4).

### County Social Determinants

There are numerous social determinants and factors that may play a role in certain differences in disease prevalence, especially for Monroe and Erie Counties. Data on income inequality, poverty status, educational attainment, healthcare access, social factors, and environmental factors for Erie and Monroe Counties in the year 2019 are summarized in Table 6, Table 7, Table 8, Table 9 and Table 10. Overall, there was no statistically significant difference in income inequality (GINI index) between Monroe and Erie Counties (*p* = 0.4684, Monroe County = 0.4684 ± 0.0125 and Erie County = 0.4607 ± 0.0097). There was also no significant difference in the percentage of people in poverty in each county aside from Asians, who had a poverty rate of 34.5% (±8.1) in Erie County (Table 6). Educational attainment for both Monroe and Erie Counties can be seen in Table 7, with Erie County tending to have a higher prevalence of high school graduates or higher educational attainment across all races. Monroe County exhibited a higher prevalence of people with a bachelor’s degree or higher across all races aside from Hispanic/Latinos (Table 7). However, there was no available data on American Indian or Alaska Native educational attainment in Monroe County. Interestingly, Table 8 highlights the differences in access to primary care, with Monroe County having 1 primary care physician (PCP) for every 960 individuals, while Erie County only has 1 PCP for every 1230 individuals. Erie County also had more racial segregation compared to Monroe County (ranked 74 vs. 63 on a scale of 0 to 100, with higher values depicting more segregation), with greater residential segregation between Black and White residents (Table 9). Environmental factors such as housing problems and air pollution in each county are included in Table 10.

**Figure 5:**
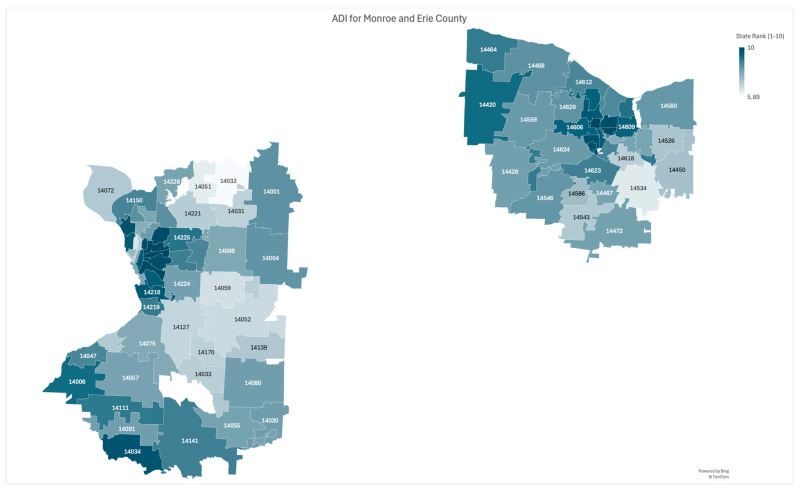
Area Deprivation Index (ADI) for Monroe County (**right**) and Erie County (**left**) based on New York State Rankings. Data retrieved from Neighborhood Atlas ADI. The five-digit numbers displayed on the map correspond to county ZIP codes. ADI is ranked from 1 to 10, with 10 being the highest ADI, or the most disadvantaged [15,16].

Area deprivation indexes for Monroe and Erie Counties are highlighted graphically in Figure 5. Both counties tended to have worse overall disparities in close proximity to the city centers. Outside suburbs, especially in specific locations such as certain regions of North and East Erie Counties, in addition to Southeast Monroe County, had better outcomes compared to the rest of the county.

## 4. Discussion

The impacts of these social determinants, especially racial segregation and healthcare access, on ocular diseases highlight the disparities that occur in modern society. Systemic societal problems and disparities lead to increased rates of underlying diseases in minority and low socioeconomic populations, such as diabetes and glaucoma. Although Erie County exhibited worse outcomes in regard to AMD and glaucoma prevalence compared to Monroe County, both counties are still affected by disparities in care and outcomes, similar to other areas around the country. Policymakers and healthcare professionals need to continue addressing social determinants to make advancements in building health equity for all people. As there are numerous categories and subcategories of social determinants of health, each of them can be targeted individually to ameliorate any disparities in healthcare. Main areas of focus could include modifiable risk factors such as healthcare access and preventive care, economic stability, employment, food security, education and health literacy, screenings, and neighborhood and community environment.

### 4.1. Economic Stability

Economic stability influences and is correlated with several factors on health, including income, employment, and food stability. Each of these social factors contributes to inequities in healthy vision. Income level is associated with visual impairment, complexity of care, and lower eye care utilization. In the UK Biobank, a study on various environmental factors on health found that low income was associated with visual impairment [18].

Additionally, Chan et al. found that those with low socioeconomic backgrounds were more likely to be affected by ocular trauma [19]. This is likely because people in these groups often work in hazardous work environments, resulting in an increased risk of eye injuries [19]. Lower household income is correlated with higher rates of incident blindness for patients with uveitis and a reduction in eye care visits [20]. Zhang et al. found that in the National Health Interview Survey, there was a lower likelihood for people with low income who had an age-related eye disease to report visiting an eye care provider (62.7%) when compared with those with high income (80.1%) [18,21].

Overall, these reports suggest that people who have lower income or economic instability are less likely to be able to afford or have access to eye care. At the county level, areas with lower income and economic instability may have a greater risk of higher visual impairment rates. To address this situation, it is beneficial to increase access to care by providing insurance coverage, medical services, and education.

Employment status is another factor that affects vision health. In the 2016 National Health Interview Survey, respondents who were employed were less likely to self-report visual difficulty than those who were looking for work or not working [18]. Furthermore, the UK Biobank study found that visual impairment was associated with an inability to work or being unemployed [18,22]. The Los Angeles Latino Eye Study also found unemployment to be an independent risk factor for visual impairment [18,23]. Hence, it is critical for counties to provide job opportunities for the ever-growing population. In addition, educational opportunities for disadvantaged populations are needed in order to expand their job search, along with the possible careers they can pursue. This way, counties are able to provide jobs that allow equitable opportunity to make a good salary, build generational wealth, maintain mental and physical health, and provide room for growth. Employment and subsequent income are key factors that prevent people from seeking treatment or healthier lifestyle and dietary options, creating a large gap in health disparities between those of high income and those of lower income. By addressing this fundamental cause, the health gap between populations can be narrowed, in addition to many other outcomes such as education and overall community wellbeing.

### 4.2. Education Access and Quality

Educational attainment is another significant factor related to social determinants of health. People with a higher level of education may have an escalated likelihood of having healthier and longer lives. Zhang et al. found in a study that participants who had not completed high school were less likely to report receiving an eye exam when compared to college graduates [21]. Furthermore, Case et al. found that there has been a decrease in life expectancy between ages 25 and 75 in the US population for those without a four-year college degree, which is true for men and women and for Black and White people [24].

To address this situation, there could be initiatives that advocate for and provide resources for those who may have limited opportunities to obtain adequate education and career training. Improving the quality of educational and extracurricular activities in marginalized areas can provide a greater variety of opportunities for students. Exposing them to mentorship opportunities, especially with diverse mentors in a wide range of fields, and providing pathway programs can allow for expanded career choices.

Disparities in health literacy, which is the ability to find, read, understand, and use health-related information, can also impact patient outcomes. Those with lower levels of health literacy have been shown to have reduced access to care and increased hospital admissions. Specifically, among glaucoma patients, lower levels of health literacy have been associated with a decreased understanding of the disease, reduced medication compliance, increased number of missed appointments, increased difficulties with eye drop administration, lower likelihood of prescription medication refills, and more advanced visual field loss [25,26,27]. Without proper health education and health literacy, people choose to forgo receiving screenings and health checkups, in addition to having poor compliance with treatment regimens, which results in worsened outcomes in the future.

To address this, patient education must be communicated at a level that is understandable across a range of health literacy levels to ensure that the patient has a comprehensive understanding of their conditions and their treatments. Patients should thoroughly understand their treatment regimen as well, including the correct dosages, the schedule to apply the medication, and the correct medication. Sleath et al. found that educating patients on how to correctly administer glaucoma drops was positively associated with treatment adherence [28]. Furthermore, having publicly funded health education programs, in addition to simply improving the quality of education for low-income populations, can improve health literacy as well [29,30].

### 4.3. Healthcare Access and Quality

Although both Monroe and Erie Counties have a lack of access to primary care professionals, the US as a whole has even worse access, especially in rural areas, with the national PCP-to-population ratio being 1330:1 (Table 8). With Erie County having fewer primary care physicians per population and having higher rates of AMD and glaucoma compared to Monroe County, it becomes essential to analyze other, more rural, areas of the US to identify any health disparities. Due to lack of access to healthcare, especially specialists such as ophthalmologists, patients go extended periods of time without receiving checkups or receive suboptimal care, resulting in progression of diseases and worse outcomes.

Rural areas commonly lack access to high-quality healthcare, including both primary care physicians for annual visits and specialists for rare or more complex conditions. To address this issue, it may be beneficial for medical schools to emphasize these geographic health disparities as part of the curriculum and encourage future medical students to consider practicing in rural areas, whether it is part-time or full-time. It also remains critical that residency programs are created in rural hospitals or have satellite clinics in rural areas as well in order to introduce workers to the area while simultaneously providing care to the inhabitants. Moreover, training programs in community health centers can be effective in allowing for further support in addressing the primary care shortage. These training programs can tailor their training composition based on the needs of the community and the availability of various health professionals such as physicians, advanced practitioners, nurses, and medical assistants [31].

Another possible solution could be to extend screenings or the utilization of mobile clinics to these areas. Furthermore, Artificial Intelligence (AI) has shown promise in this area. For instance, AI has already been tested to be incorporated into the diagnosis of diabetic retinopathy. If the use of AI becomes more widespread in healthcare, it can be integrated into the screening process for a wide variety of diseases [32]. Utilizing AI in the diagnostic processes could potentially help ameliorate the disparities faced by these communities. For example, in the case of diabetic retinopathy, AI can be used to analyze fundus photos and determine if the patient is at risk for the disease and, as a result, determine if that person needs to be referred to a specialist.

### 4.4. Neighborhood and Built Environment

The environments where people live have a critical role in their health and wellbeing, including vision outcomes and access to eye care. In a study conducted in Sweden, higher levels of neighborhood deprivation, which were measured using factors such as income level, employment status, education level, and receipt of social welfare, were associated with increased risk of age-related eye diseases [33].

Historical redlining, the systematic denial of mortgages or loans to residents in a certain location with the intent to create segregation, resulted in underfunded minority communities being located in less desirable and unsafe areas, with added health risks [34]. The location of communities can influence access to primary care, which can help decrease the rates of diseases through increased prevention screenings [34]. Unfortunately, many of these redlined communities lack access to various necessities, including primary care. As seen in Figure 5, communities that were redlined and more diverse tended to be more disadvantaged compared to neighborhoods that were primarily White.

One way to address this would be to improve construction or renovation in neighborhoods that are heavily affected by this issue. This is especially true for densely packed areas such as urban environments. Initiatives aimed at maintaining adequate and safe housing is another possible aspect of the solution. Factors such as employment location, safety, or socioeconomic status might result in people living in these neighborhoods. However, the state of the neighborhood and built environment can be addressed to ensure residents are not in unsafe conditions, such as pollution, mold, or asbestos exposure. Furthermore, policy changes can be implemented to prevent aspects such as pollution from surrounding factories or other sources.

Another way the environment that people live in impacts community health is through access to healthy foods. According to the Economic Research Service of the U.S. Department of Agriculture, approximately 13.5% of households in the United States experience food insecurity. Food insecurity is defined as limited access to nutritious foods and has been associated with chronic diseases, such as diabetes and hypertension, as well as ocular diseases and self-reported visual impairment in both the NHIS and the National Health and Nutrition Examination Survey. Lack of nutrition and the stress that can be associated with it may lead to physiological effects that eventually result in vision loss [35,36]. Therefore, both the availability and cost of food in a community could impact the overall wellbeing of those residing within it. An overarching goal to address this issue is to increase access to less expensive and healthy food options through promoting construction of supermarkets that have fresh, unpackaged produce or farmers’ markets with fresh, healthy options.

### 4.5. Social and Community Context

Interactions with your social network, including family, friends, and coworkers, can further affect health, especially in regard to race and identity. Members of Black populations have a higher incidence of emergency department encounters for visual impairment compared to White populations. In a study of 78,526 participants with glaucoma, Halawa et al. found that in both low and non-low-socioeconomic groups, Black people had lower outpatient visits and visual field testing and higher rates of inpatient or emergency department visits [37]. The relationship of race and health outcomes is complex but is highly prevalent in all aspects of healthcare, including eye care [18].

Part of the reason for the current relationship between race and health outcomes is likely due to implicit bias. Implicit biases are internalized attitudes or stereotypes that may unconsciously impact our perceptions, actions, and decisions. It is another factor that has long contributed to health disparities. For instance, when caring for marginalized patients, the provider’s bias influences communication with the patient, potentially resulting in suboptimal decision-making. The patient may sense the bias, may distrust the provider and system, and may decide not to follow through on treatment plans or may modify them. Racism, sex and gender discrimination, and other forms of discrimination must be eliminated, as they prevent marginalized trainees and faculty from thriving, create stereotype threat for the marginalized, and confirm bias for the non-marginalized [38,39].

To address implicit bias in healthcare, it is recommended that healthcare workers partake in implicit bias training. Although there is still limited knowledge and specific effective methods on how to reduce implicit bias, various studies have provided suggestions. For instance, strategies such as incorporating mindfulness, coalition-building, and personal retrospection have been recommended to reduce the harmful effects of implicit bias in the medical environment. More specifically, to reduce implicit racial bias, methods can include finding common things between each other or expanding your social network and forming friendships with people of different healthcare professions [40].

Cultural competency and humility are also skills that healthcare workers should harness since cultural misunderstandings and miscommunications can negatively impact patient health outcomes, in addition to deterring minority populations from seeking healthcare. Cultural humility refers to an orientation toward providing care based on self-assessment, appreciation of the patient’s expertise on social and cultural contexts of their lives, willingness to establish power-balanced relationships with patients, and being a lifelong learner [41]. While the term “competency” implies mastery, humility can signify inter- and intra-personal tactics in individualized care. By promoting self-reflection, healthcare providers can acknowledge that there is always more to learn from a patient, which can assist in creating more patient-centered care [41]. By having the workforce trained in respecting a patient’s culture and beliefs, the healthcare environment can become more inclusive toward minority groups, encouraging them to seek support and build trust with providers.

### 4.6. Limitations

There are some potential limitations to this study. Although the VEHSS database provides valuable insights into disease prevalence across various counties and regions nationwide, the sole reliance on Medicare data might not allow for an accurate representation of the entire population of the county. Additionally, although 2019 was chosen for the stratification of prevalence data, ADI data was not available in 2019. The year 2022 was chosen because the closest available ADI data was for 2020, which was significantly impacted by the COVID-19 pandemic. These results cannot be interpreted on an individual level because this was an ecological study comparing two counties. Hence, future research should expand on these findings by targeting whether there are associations between certain variables and disease prevalence. Future studies should also analyze data after 2022 to increase applicability to modern-day public health problems.

## 5. Conclusions

Health disparities significantly affect minority groups in both vision and healthcare in general. Although this study targeted Western New York, lack of healthcare access and disparities caused by historic redlining are national problems. Erie County had an increased prevalence of AMD and glaucoma compared to Monroe County in 2019. Certain diseases and conditions, such as glaucoma, diabetes, and hypertension, are more prevalent among minority populations, a difference that may be attributed to socioeconomic circumstances. To potentially ameliorate these health inequities, it is important to focus on social determinants of health and underlying systemic problems that increase the burden on minority populations. Addressing these factors is a complex task, and it requires interdisciplinary approaches, ranging from overarching strategies such as policy development and changes to individualized methods such as implicit bias training. There should be an emphasis on holistic care, where a patient’s circumstances are taken into account, including their culture, religion, social capital, and residential area. Schools, education programs, and educators should be prepared to provide training on the social determinants of health in order to foster diverse environments in various sectors. Creating a diverse healthcare environment with trained doctors will allow more people of color to feel comfortable and cared for. In addition to increasing trust in the healthcare system, this will lead to better mental health and productivity of the workers and the patients as well. Every person should have access to high-quality healthcare, which includes preventive and screening programs. To reach this goal, it is necessary that all people involved, including healthcare workers, government officials, and administrators, among many stakeholders, participate in the effort to increase healthcare access for all patients. While moving forward and finding innovative solutions to improve the health outcomes of populations affected by the social determinants of health, it remains crucial to prioritize and remember the overarching goal of reforming the systemic problems that disadvantaged populations face. Every individual will need to be involved to shift the current paradigm and systemic problems that affect minorities and disadvantaged populations. By addressing the underlying problem of lack of resources, systemic racism, and segregation, a trickle-down effect could be seen, where certain social determinants are less prevalent in the population, resulting in a systemic improvement of health outcomes, including ocular health. All have to be vested, including patients, to make a change to the current system.

## Figures and Tables

**Table 1:** Ocular disease crude prevalence in Erie and Monroe Counties in 2019. Data is provided as prevalence (%) with 95% confidence intervals. Erie County sample size = 49,300. Monroe County sample size = 32,500.

Disease	Prevalence (%)		*p*-Value
	Erie County	Monroe County	
AMD	9.56 (9.30–9.82)	6.61 (6.34–6.88)	<0.0001
Diabetic Retinopathy	2.97 (2.83–3.13)	3.01 (2.83–3.21)	0.74
Glaucoma	13.05 (12.76–13.35)	11.71 (11.36–12.06)	<0.0001

**Table 2:** Ocular disease crude prevalence in the US and New York State in 2019. Data is provided as prevalence (%) with 95% confidence intervals. US sample size = 29,607,800. New York State (NYS) sample size = 1,549,100.

Disease	Prevalence (%)	
	USA	New York State
AMD	9.37 (9.36–9.38)	11.18 (11.13–11.23)
Diabetic Retinopathy	3.11 (3.10–3.11)	3.55 (3.52–3.58)
Glaucoma	13.60 (13.59–13.61)	19.00 (18.94–19.06)

**Table 3:** Ocular disease prevalence in Erie and Monroe Counties based on race in 2019. Data is provided as prevalence (%) with 95% confidence intervals.

Disease	Race	Erie County		Monroe County		*p*-Value
		Sample Size	Prevalence (%)	Sample Size	Prevalence (%)	
AMD	Asian	730	8.13 (6.24–10.36)	780	6.27 (4.68–8.21)	0.16
	Black	7000	2.44 (2.09–2.83)	5300	1.40 (1.11–1.76)	<0.0001
	Hispanic	1500	3.34 (2.50–4.37)	2100	2.95 (2.27–3.76)	0.51
	Native	130	3.13 (0.86–7.81)	30	8.82 (1.86–23.68)	0.29
	White non-Hispanic	39,500	11.15 (10.84–11.46)	23,900	8.13 (7.79–8.49)	<0.0001
	Other	430	6.54 (4.39–9.32)	290	4.20 (2.19–7.21)	0.16
DR	Asian	730	3.86 (2.58–5.53)	780	4.48 (3.14–6.18)	0.55
	Black	7000	4.76 (4.28–5.29)	5300	4.68 (4.13–5.28)	0.84
	Hispanic	1500	4.78 (3.77–5.98)	2100	4.85 (3.97–5.85)	0.92
	Native	130	8.59 (4.37–14.86)			
	White non-Hispanic	39,500	2.54 (2.39–2.70)	23,900	2.41 (2.22–2.61)	0.31
	Other	430	3.97 (2.33–6.28)	290	4.90 (2.70–8.08)	0.56
Glaucoma	Asian	730	15.84 (13.26–18.70)	780	9.35 (7.40–11.61)	0.0001
	Black	7000	15.08 (14.25–15.94)	5300	12.68 (11.80–13.60)	0.0001
	Hispanic	1500	10.62 (9.11–12.27)	2100	9.22 (8.01–10.53)	0.17
	Native	130	6.25 (2.74–11.94)	30	11.76 (3.30–27.45)	0.38
	White non-Hispanic	39,500	12.77 (12.44–13.10)	23,900	11.81 (11.41–12.23)	0.0003
	Other	430	12.15 (9.21–15.63)	290	9.44 (6.31–13.44)	0.24

**Table 4:** Ocular disease prevalence in Erie and Monroe Counties based on gender in 2019. Data is provided as prevalence (%) with 95% confidence intervals.

Disease	Gender	Erie County		Monroe County		*p*-Value
		Sample Size	Prevalence (%)	Sample Size	Prevalence (%)	
AMD	Male	22,700	7.42 (7.08–7.77)	14,800	4.99 (4.65–5.36)	<0.0001
AMD	Female	26,700	11.39 (11.01–11.77)	17,600	7.96 (7.57–8.37)	<0.0001
DR	Male	22,700	3.12 (2.9–3.36)	14,800	3.23 (2.95–3.53)	<0.0001
DR	Female	26,700	2.85 (2.65–3.05)	17,600	2.83 (2.59–3.08)	0.90
Glaucoma	Male	22,700	11.79 (11.37–12.22)	14,800	10.75 (10.25–11.26)	0.002
Glaucoma	Female	26,700	14.13 (13.71–14.55)	17,600	12.52 (12.03–13.01)	<0.0001

**Table 5:** Ocular disease prevalence in Erie and Monroe Counties based on age in 2019. Data is provided as prevalence (%) with 95% confidence intervals.

Disease	Age	Erie County		Monroe County		*p*-Value
		Sample Size	Prevalence (%)	Sample Size	Prevalence (%)	
AMD	18–39			2300	0.26 (0.09–0.56)	
	40–64	11,000	1.17 (0.98–1.39)	8400	0.77 (0.60–0.99)	0.004
	65–84	29,700	9.50 (9.16–9.84)	17,500	6.39 (6.03–6.76)	<0.0001
	85+	6200	28.44 (27.32–29.59)	4300	22.51 (21.26–23.79)	<0.0001
DR	18–39	2400	0.97 (0.61–1.45)	2300	1.12 (0.73–1.63)	0.61
	40–64	11,000	3.18 (2.86–3.52)	8400	3.60 (3.21–4.02)	0.11
	65–84	29,700	3.15 (2.96–3.36)	17,500	3.29 (3.03–3.57)	0.41
	85+	6200	2.53 (2.15–2.95)	4300	1.74 (1.37–2.18)	0.01
Glaucoma	18–39	2400	2.74 (2.12–3.47)	2300	2.28 (1.71–2.97)	0.31
	40–64	11,000	6.21 (5.77–6.68)	8400	6.31 (5.80–6.85)	0.78
	65–84	29,700	15.20 (14.79–15.61)	17,500	13.78 (13.27–14.30)	<0.0001
	85+	6200	18.92 (17.95–19.92)	4300	19.03 (17.86–20.24)	0.89

**Table 6:** Percent poverty status for Monroe and Erie Counties in 2019. N = No data available.

Percent of Population Below Poverty Level	Monroe County	Erie County	*p*-Value
All ages, all genders, all races	12.7%± 1.0	13.3% ± 1.0	0.67
Male	12.3% ± 1.2	12.1% ± 0.9	0.89
Female	13.1% ± 1.2	14.4% ± 1.3	0.46
Asian	7.7% ± 4.6	34.5% ± 8.1	<0.0001
Black, non-Hispanic	25.1% ± 4.0	27.9% ± 4.8	0.65
Hispanic, any race	25.7% ± 5.4	32.3% ± 5.7	0.40
North American Native	N	N	N
White, non-Hispanic	8.2% ± 1.1	8.0% ± 0.8	0.88

**Table 7:** Education Attainment in Monroe and Erie Counties in 2019. Total estimate = estimate for population in the county. N = No data available.

Educational Attainment, 2019	Erie County			Monroe County		
Label	Total Estimate	Estimate (%)	Margin of Error	Total Estimate	Estimate (%)	Margin of Error
**White alone**	527,571			409,987		
High school graduate or higher	501,883	95.1	0.4	383,873	93.6	0.7
Bachelor’s degree or higher	190,799	36.2	1.2	182,820	44.6	1.3
**White alone, not Hispanic or Latino**	515,388			387,435		
High school graduate or higher	490,955	95.3	0.4	367,556	94.9	0.6
Bachelor’s degree or higher	187,617	36.4	1.2	177,782	45.9	1.3
**Black alone**	77,194			70,855		
High school graduate or higher	66,757	86.5	2.3	57,438	81.1	3
Bachelor’s degree or higher	13,696	17.7	2.9	12,876	18.2	2.6
**American Indian or Alaska Native alone**	3757			N		
High school graduate or higher	3344	89	6.9	N	N	N
Bachelor’s degree or higher	691	18.4	11.4	N	N	N
**Asian alone**	19,942			16,297		
High school graduate or higher	16,126	80.9	4.1	12,556	77	7.6
Bachelor’s degree or higher	10,039	50.3	6.8	9091	55.8	8.8
**Hispanic or Latino Origin**	28,216			37,193		
High school graduate or higher	22,221	78.8	5	27,019	72.6	4
Bachelor’s degree or higher	6360	22.5	4.7	7892	21.2	4.2

**Table 8:** Healthcare access metrics for Monroe and Erie Counties, New York State (NYS), and the US in 2019. Uninsured: percentage of population under age 65 without health insurance. Other Primary Care Providers: nurse practitioners (NPs), physician assistants (PAs), and clinical nurse specialists who can provide routine and preventive care [13].

Healthcare Access 2019	Erie County	Monroe County	NYS	US
Uninsured	5%	5%	7%	10%
Primary Care Physicians Ratio to Population	1230:1	960:1	1200:1	1330:1
Other Primary Care Providers	688:1	527:1	944:1	1133:1

**Table 9:** Social Factors in Monroe and Erie Counties, New York State (NYS), and the US in 2019. Residential Segregation Black/White: index of dissimilarity where higher values indicate greater residential segregation between Black and White county residents. Food Environment Index: index of factors that contribute to a healthy food environment, from 0 (worst) to 10 (best) [13].

Social Factors 2019	Erie County	Monroe County	NYS	US
Adult Smoking (% current smokers)	18%	16%	14%	
Adult Obesity % Adults (≥20 yrs)	30%	30%	26%	29%
Food Environment Index (0 to 10)	7.9	7.9	9.1	7.7
% Adults (≥20 yrs) Reporting No Physical Inactivity	25%	23%	25%	22%
% Population with Access to Exercise Opportunities	96%	95%	93%	84%
Residential Segregation Black/White (0 to 100)	74	63	74	

**Table 10:** Environmental Factors in Monroe and Erie Counties, New York State (NYS), and the US in 2019. Air Pollution—Particulate Matter: average daily density of fine particulate matter in micrograms per cubic meter (PM2.5). Severe Housing Problems Definition: percentage of households with at least 1 of 4 housing problems: overcrowding, high housing costs, lack of kitchen facilities, or lack of plumbing [13].

Environmental Factors 2019	Erie County	Monroe County	NYS	US
Air Pollution—Particulate Matter	10.7	8.9	8.5	
Severe Housing Problems	15%	17%	24%	18%

## Data Availability

The data presented in the study are openly available in American Community Survey (ACS) at https://www.census.gov/programs-surveys/acs.html (accessed on 11 November 2024), County Health Rankings & Roadmaps at https://www.countyhealthrankings.org/health-data/new-york?year=2025 (accessed on 11 November 2024), Vision and Eye Health Surveillance System (VEHSS) at https://www.cdc.gov/vision-health-data/index.html (accessed on 11 November 2024), and Neighborhood Atlas at https://www.neighborhoodatlas.medicine.wisc.edu (accessed on 11 November 2024).
